# Rhinocerebral mucormycosis: A 15-year retrospective study in southern Spain

**DOI:** 10.4317/medoral.27986

**Published:** 2026-03-07

**Authors:** Lucas Fernandez-Figares-Conde, Inmaculada Isorna, Eusebio Torres-Carranza, Alberto Garcia-Perla-Garcia, Angel Rollon-Mayordomo, Pedro Infante-Cossio

**Affiliations:** 1 Department of Oral and Maxillofacial Surgery, Virgen del Rocio University Hospital, Seville, Spain; 2 Department of Otorhinolaryngology, Virgen del Rocio University Hospital, Seville, Spain; 3 Department of Surgery, School of Medicine, University of Seville, Seville, Spain; 4 Department of Oral and Maxillofacial Surgery, Virgen Macarena University Hospital, Seville, Spain

## Abstract

**Background:**

Rhinocerebral mucormycosis (RCM) is a severe, rapidly progressing opportunistic fungal infection with a high mortality rate, primarily affecting immunocompromised patients with diabetes mellitus or haematological malignancies. Its incidence has increased in recent years, particularly since the SARS-CoV-2 pandemic, coinciding with population ageing and the growing prevalence of immunocompromised patients. Data regarding survival rates and the most effective diagnostic and therapeutic strategies in Spain are limited. This study analyses the clinical characteristics, risk factors, and prognosis of RCM at a tertiary care centre in southern Spain.

**Material and Methods:**

A retrospective study was conducted on 36 patients treated for RCM between 2009 and 2023. Clinical-epidemiological variables, history of immunosuppression, extent of infection, diagnostic and therapeutic management, and survival outcomes were evaluated. Statistical analysis was performed using chi-square tests and regression models (p&lt;0.05).

**Results:**

Males predominated (66.7%), with a mean age of 58 years (range 17-83). The primary risk factors were haematological malignancies (61.1%), solid tumours (16.6%), and uncontrolled diabetes mellitus (22.2%). The most common clinical presentation was orbital involvement (86.1%), followed by sinusitis (47.2%), neurological symptoms (36.1%), and palatal necrosis (25%). Imaging studies revealed pansinusitis in 97.2% of cases and periorbital/nasomaxillary cellulitis in 66.7%. Treatment consisted of combined antifungal therapy (liposomal amphotericin B/azoles) and management of the underlying disease. Twenty-seven patients (75%) underwent surgical intervention, including endoscopic sinus surgery, maxillectomy, or orbital exenteration. Overall mortality was 66.7% (24 deaths), which was significantly associated with advanced age and the absence of surgical treatment.

**Conclusions:**

RCM is a rare but highly lethal infection in our setting. Early diagnosis and prompt combined treatment, involving multidisciplinary management and standardised protocols, are essential to improve outcomes. The main prognostic factors identified were age, control of immunosuppression, and timely surgical intervention. Further multicentre studies are needed to optimise treatment strategies.

## Introduction

Rhinocerebral mucormycosis (RCM) is a rare, invasive, opportunistic fungal infection with high mortality rates and a rapid clinical progression (1). In developing countries, the disease predominantly affects immunocompromised patients with poorly controlled diabetes mellitus (DM), whereas in developed regions, it is more frequently associated with haematological malignancies and transplant recipients (2 , 3). In recent years, the incidence of RCM has escalated significantly, particularly following the global SARS-CoV-2 pandemic. This trend is likely driven by the widespread use of broad-spectrum antibiotics and high-dose corticosteroids, coinciding with population ageing and the increasing number of immunocompromised patients (4 , 5). Currently, mucormycosis ranks as the third most common cause of invasive fungal infection, after aspergillosis and candidiasis (1), with RCM representing its most frequent clinical presentation (6).

Early diagnosis of RCM remains challenging due to the non-specific nature of initial symptoms and the rapid progression of the infection. The typical clinical presentation follows the inhalation of fungal spores, leading to angioinvasion of the rhinosinusal and orbital tissues. Patients often present with orbital cellulitis, ophthalmoplegia, sinusitis, and necrotic eschars on the oral mucosa, particularly on the palate. Intracranial extension may result in an altered level of consciousness or complications such as cavernous sinus thrombosis (7 , 8). Early identification through histopathological, microbiological and imaging studies is pivotal in minimising diagnostic delays and facilitating prompt treatment, thereby optimising prognosis (9).

Although the incidence of mucormycosis remains relatively low in Spain compared to other regions, a marked increase has been recorded in recent decades. Between the periods 1988-2006 and 2007-2015, the incidence rate rose from 1.2 to 3.3 cases per 100,000 hospital admissions (10). Similarly, Parra Fariñas et al. reported a significant increase in the annual incidence rate, rising from 0.74 to 1.24 cases per million person-years over the last two decades (11). Given the rising morbidity and mortality burden associated with RCM, a multidisciplinary approach and the implementation of institutional protocols at referral centres are warranted. While RCM is the most common form of mucormycosis, data regarding survival rates and optimal diagnostic and therapeutic strategies within the Spanish healthcare system remain limited (12). Therefore, this study aimed to analyse the clinical and epidemiological characteristics, risk factors, and prognostic indicators in patients diagnosed with RCM at a tertiary care hospital in southern Spain. The primary objective was to identify factors associated with patient survival and evaluate therapeutic outcomes.

## Material and Methods

Study design and patient selection

A retrospective clinical study was conducted at the Virgen del Rocio University Hospital in Seville, a tertiary referral centre for infectious diseases and haematological malignancies in southern Spain. The study population comprised all patients with confirmed RCM of the maxillofacial region, diagnosed using microbiological and/or histopathological criteria, who were treated in the Departments of Oral and Maxillofacial Surgery and Otolaryngology between 2009 and 2023. Patients treated at external centres or those presenting with mucormycosis localised outside the head and neck region were excluded. The study protocol was approved by the institutional Research Ethics Committee (internal code: 2024-001205).

Data collection

Demographic and clinical data, including age, gender, underlying comorbidities, history of immunosuppression, initial clinical presentation and disease progression, were retrieved for all patients. Imaging studies (CT and MRI) were examined to determine the anatomical location and extent of the infection. Details of antifungal regimens, the management of comorbidities and the type of surgical interventions were also recorded. Clinical outcomes, including therapeutic response, complications and mortality, were monitored from hospital admission onwards.

Multidisciplinary management protocol

In accordance with our institutional Diagnostic and Therapeutic Guidelines (https://www.guiaprioam.com/indice/abordaje-terapeutico-mucormicosis-en-adulto-protocolo-hvr/), biopsies of the oral cavity (performed by the Department of Oral and Maxillofacial Surgery) and the sinonasal region (performed by the Department of Otolaryngology) were obtained upon clinical suspicion of RCM. Fresh tissue samples were collected for microbiological culture, while formalin-fixed specimens were submitted for histopathological analysis. Antifungal therapy with liposomal amphotericin B was initiated immediately at a dose of 5 mg/kg/day, escalating to 10 mg/kg/day in patients with suspected central nervous system involvement or history of transplantation.

Subsequently, following imaging assessment (CT/MRI) and microbiological confirmation, a personalised pharmacological and/or surgical treatment plan was established through coordination between the Departments of Infectious Diseases, Oral and Maxillofacial Surgery, and Otolaryngology. Follow-up care was conducted in collaboration with the Departments of Endocrinology and Haematology, assessing immunological status and monitoring daily clinical progress tailored to each patient's underlying pathology. Antifungal management consisted of advanced-generation agents combined with azoles in selected cases for salvage or maintenance therapy. Supportive measures were implemented, such as strict glycaemic control for diabetic patients and the use of haematopoietic growth factors for patients with haematological malignancies.

Surgical management involved techniques ranging from minimally invasive endoscopic procedures to extensive resections, such as maxillectomy, orbital exenteration, or craniotomy. The surgical approach was determined sequentially by multidisciplinary consensus, considering disease stage, extent of infection, prognostic factors, comorbidities, and individual clinical status. The primary surgical objective was to achieve complete debridement with negative margins.

Statistical analysis

Categorical variables were expressed as absolute frequencies and percentages, while continuous variables (age and follow-up duration) were described using means, standard deviations (SD), and ranges. The Pearson chi-square test was employed to evaluate associations between categorical prognostic factors. The impact of age on mortality was analysed using Cox regression modelling. Statistical significance was set at p&lt;0.05.

## Results

**Figure 1:**
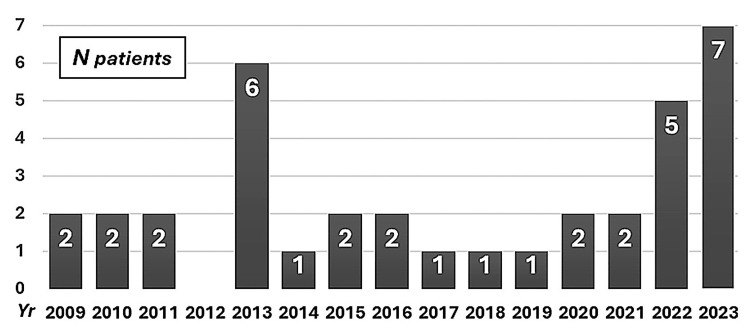
Temporal distribution of cases (2009-2023).

A total of 36 patients were included in the study, comprising 24 males (66.7%) and 12 females (33.3%), with a mean age of 58 years (range 17-83 years). Analysis of the annual incidence revealed peaks in 2013, 2022 and 2023, reaching a maximum of seven cases per year (Figure 1).

1

Haematological malignancies were the predominant underlying condition, affecting 22 patients (61.1%) with acute leukaemia being the most frequent (16 cases, 44.4%), followed by lymphoma (4 cases, 11.1%) and multiple myeloma (2 cases, 5.5%). Six patients (16.6%) presented with immunosuppression and neutropenia associated with solid tumours (three lung cancers, one anal cancer, and one bladder cancer) or liver transplantation for hepatocellular carcinoma (one case). Uncontrolled DM was identified in eight patients (22.2%), including two with type 1 DM and six with type 2 DM. Additionally, eight patients (22.2%) were recipients of haematopoietic stem cell transplantation, and five (13.9%) suffered from chronic kidney disease.

Table 1 summarizes the clinical and radiological features of the cohort.

**Figure 2:**
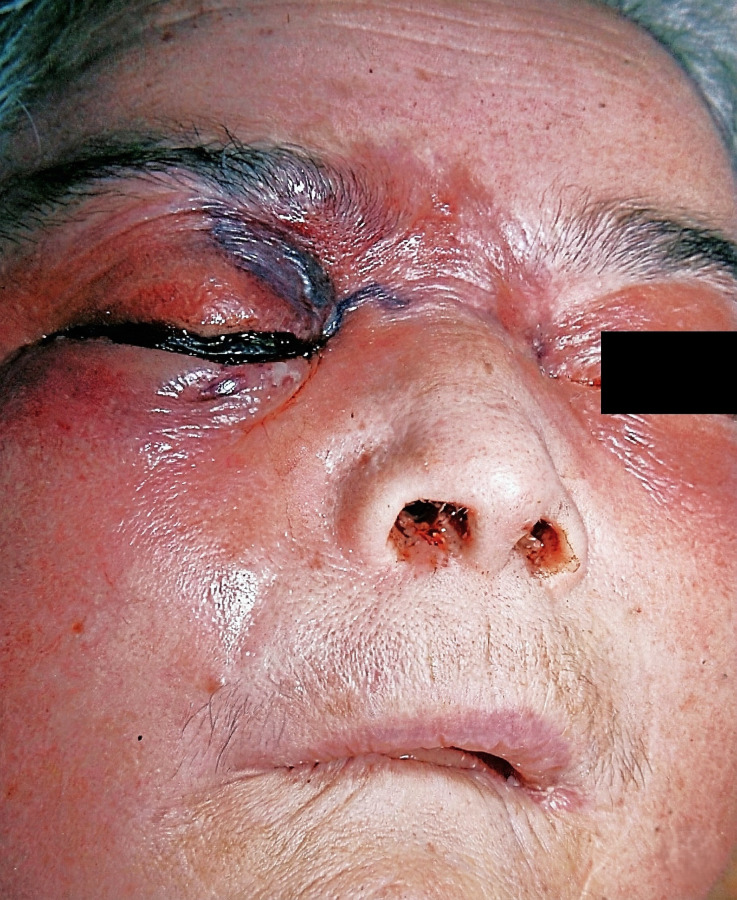
Cutaneous necrosis and periorbital cellulitis in a patient with uncontrolled DM.

**Figure 3:**
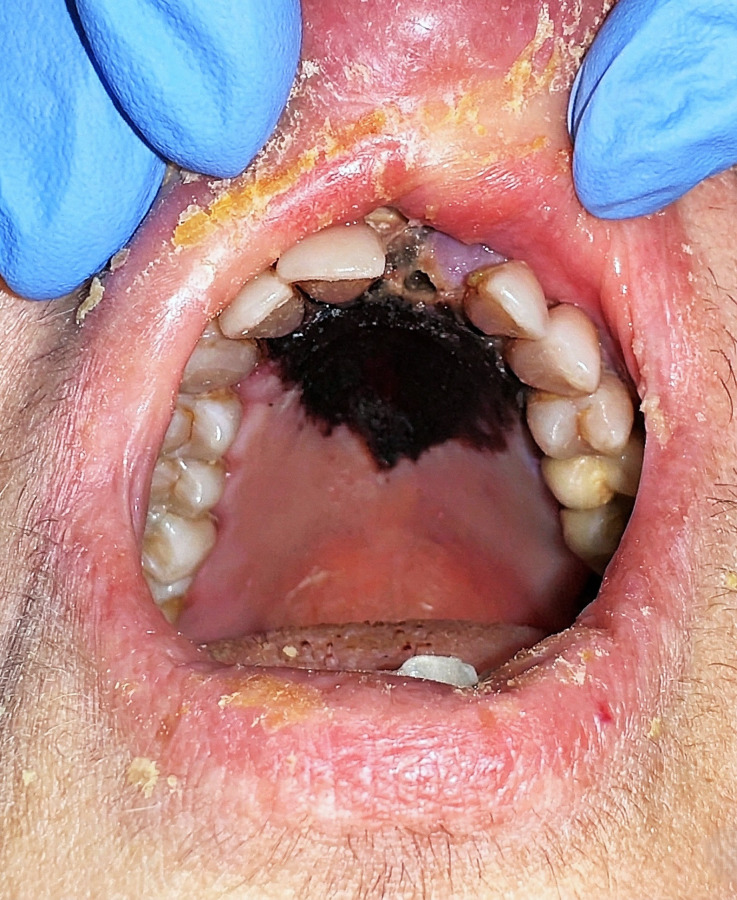
Black eschar on the palate in a patient with acute leukaemia.

Table 1The clinical presentation exhibited four principal patterns, highlighting the aggressive and multifocal nature of this opportunistic infection (Figure 2 and Figure 3). Orbital involvement was the most frequent clinical manifestation, characterised by periorbital cellulitis or ophthalmoplegia in 31 patients (86.1%). All patients underwent an initial assessment using CT scanning, and five required additional MRI scans to evaluate intracranial and soft tissue extension. The most consistent radiological finding was pansinusitis, which was observed in 35 patients (97.2%).

2

3

All confirmed cases were diagnosed based on microbiological findings. Rhizopus spp. was the most frequently identified genus, accounting for 30 cases (83.3%), of which Rhizopus arrhizus was present in nine cases. This was followed by Mucor spp. (5 cases, 13.9%) and Cunninghamella (1 case, 2.8%). Histopathological examination revealed compatible fungal hyphae and angioinvasion in 32 patients (88.9%).

All patients received intravenous liposomal amphotericin B, administered as monotherapy in 18 cases and in combination therapy in the remaining 18, notably including isavuconazole in 11 cases. The maximum dose was 5 mg/kg in 25 patients, 7.5 mg/kg in two, and 10 mg/kg in nine, under strict electrolyte monitoring. The mean duration of intravenous treatment was 24.9 days (range 5-90 days). Ten patients transitioned to oral therapy, receiving either posaconazole (6 cases) or isavuconazole (4 cases). Supportive care included intensive insulin therapy with strict glycaemic control for diabetic patients and granulocyte colony-stimulating factor (G-CSF) administration in twelve patients. In terms of management, nine patients (25%) were treated with antifungal therapy alone, while 27 patients (75%) underwent surgical debridement in addition to medical therapy.

The overall mortality rate was 66.7% (24 deaths), with a mean survival time from diagnosis to death of 15.8 days (range 3-36 days). A statistically significant difference in mortality was observed based on treatment modality: Patients who underwent surgery had a mortality rate of 55.6% (15/27), compared to 100% (9/9) in those treated exclusively with medical therapy (p&lt;0.001) (Table 2). Cox regression analysis identified age as an independent prognostic factor (p=0.047), with a hazard ratio of 1.039 (95% CI: 1.001-1.078), indicating a 3.9% increase in mortality risk for each additional year of age.

Table 2Univariate analysis of prognostic factors revealed that advanced age (&gt;55 years) was the only variable significantly associated with higher mortality (p=0.020) (Table 3).

Table 3No significant differences in survival were found regarding gender, presence of haematological malignancies, solid tumours, or uncontrolled DM.

Among the 27 surgically treated patients, endoscopic sinus surgery (ESS) was the most common approach, performed in 22 cases (81.5%) and serving as the sole intervention in eight (29.6%). More extensive procedures included maxillectomy combined with ESS (9 cases, 33.3%), orbital exenteration/nasal amputation combined with ESS (3 cases, 11.1%), and radical resection involving maxillectomy, orbital exenteration, and craniotomy combined with ESS (7 cases, 19.4%). No statistically significant difference in mortality was observed across the different surgical techniques (p=0.858). However, a trend towards lower mortality was noted in the orbital exenteration/nasal amputation group (33.3%), whereas the highest mortality was recorded in patients treated with ESS alone (62.5%) (Table 4).

Table 4

## Discussion

Our study of 36 patients with RCM, evaluated over a 15-year period, corroborates the rarity of this invasive fungal infection in Spain. The rising incidence observed in recent years, particularly in 2022 and 2023 with seven and five cases, respectively, mirrors epidemiological trends reported in developed nations, in contrast to disease distribution patterns in developing regions (13). The male predominance observed in our cohort (66.7%) is consistent with existing literature, although the underlying mechanisms for this gender-based predisposition remain to be elucidated (14). Notably, the mean age of 58 years in our series substantially exceeded the 42 years reported in the systematic review by Kumar et al. (14). From a prognostic standpoint, this finding is significant, as our analysis indicates that each additional year of age increases mortality risk by 3.9%, a critical factor for the initial prognostic assessment.

The risk profile identified in our study differs from that reported in countries such as India, Iran, or Mexico, where uncontrolled DM was the predominant predisposing factor for RCM (15 - 17). In our series, 28 patients (77.8%) presented with haematological malignancies or immunosuppression associated with solid tumours, a pattern consistent with developed countries (18). Although therapeutic advances have prolonged survival in these patient population, they have concurrently increased the prevalence of neutropenia and sustained immunosuppression. While no statistically significant difference in survival was found regarding uncontrolled DM, a trend towards better outcomes was observed in the diabetic subgroup (n=8, 22.2%). This may reflect greater awareness of glycaemic control in our setting, although RCM remains a serious threat to young patients with type 1 diabetes and poor treatment adherence. Interestingly, Nezafati et al. identified tooth extraction in poorly controlled DM patients as a specific trigger for RCM (19). Another distinctive finding in our study was the presence of immunosuppressed patients with solid tumours, as well as a liver transplant recipient. This group is rarely described in the literature as being at primary risk of RCM (14 , 20 - 22).

The symptomatology in our study generally aligns with established patterns. Orbital manifestations (86.1%) predominantly presented as periorbital cellulitis or ophthalmoplegia accompanied by the "frozen eye" sign, which is consistent with previous studies (8). Paranasal sinusitis (47.2%) was a frequent early manifestation, whereas palatal necrosis (25%) was less common, being associated with advanced disease and a poor prognosis (23). Neurological involvement (36.1%) typically manifested as non-specific symptoms such as somnolence, altered consciousness and frontal lobe signs, which were attributable to local inflammation or cavernous sinus thrombosis. In contrast, Sharma et al. reported a high incidence of frontotemporal abscesses in India, with a mortality rate of 42.6%. This discrepancy may be explained by earlier diagnosis and intervention in our setting, which likely mitigates progression to extensive intracranial abscess formation.

Radiological assessment via CT scan revealed pansinusitis in 97.2% of cases and periorbital or nasomaxillary cellulitis in 66.7%. The prognosis worsens considerably when the paranasal sinuses, bone and orbit are simultaneously involved. Notably, bone erosion was detected in only 8.3% of cases. Slonimsky et al. (21) have emphasised the limited sensitivity of CT in detecting early bone erosion in RCM. This finding calls into question the usefulness of bone erosion as the sole criterion for surgical decision-making and highlights the importance of a complementary endoscopic assessment to optimise therapeutic planning. MRI was found to be superior for evaluating intracranial extension due to possible vascular and perineural invasion (14).

First-line antifungal therapy in our protocol was intravenous liposomal amphotericin B, the gold standard due to its superior efficacy and toxicity profile compared to other formulations. In selected cases, sequential or combination therapy with azoles (isavuconazole or posaconazole) was employed, highlighting the emerging role of posaconazole as a maintenance agent (24 , 25). Zhang et al. have suggested that, in specific scenarios, a combination of posaconazole and conservative surgery could be a viable alternative for patients with cavernous sinus invasion, although clinical evidence remains limited (24).

Effective management of RCM requires an urgent, multidisciplinary approach based on three pillars: Reversal of immunosuppression, early antifungal therapy, and surgical debridement. Regarding control of the underlying disease, DM patients showed a trend towards better survival, suggesting that restoring immunological competence is a key prognostic factor, as noted by Martínez-Herrera et al. (16) and Abu El-Naaj et al. (20). In clinical practice, strategies such as intensive insulin therapy and G-CSF administration for neutropenia are critical for improving survival.

In our study, surgical intervention was found to be a decisive factor for survival. A statistically significant difference in mortality was observed between patients who underwent surgery and those who were treated with antifungals alone (55.6% vs. 100%, p&lt;0.001). This underscores the importance of surgery within a comprehensive treatment scheme. Although no significant difference was found between surgical techniques (p=0.858), clinically relevant trends emerged. Patients treated with ESS alone had a mortality rate of 62.5%, whereas those undergoing maxillectomy, orbital exenteration or craniotomy had a rate of 57.1%. This apparent paradox can be explained by the use of ESS in two distinct scenarios: In cases of early localised disease, where it was curative in three survivors with infection limited to sinusitis, and in critically ill patients who were unfit for aggressive surgery due to severe comorbidities or persistent neutropenia. Zhang et al. (24) reported outcomes on endoscopic debridement without orbital exenteration, suggesting that conservative surgery may suffice for carefully selected patients with localised disease, early diagnosis, and adequate metabolic control. These findings support our institutional protocol, which advocates a progressive, sequential surgical strategy tailored to clinical evolution and patient status.

An unexpected finding was the absence of mortality among the four patients with intracranial extension who underwent surgery. Three of the patients presented with cavernous sinus thrombosis, while the fourth presented with an inaccessible extra-axial abscess that resolved following combined treatment. This contrasts with the 42.6% mortality rate reported by Sharma et al. (8) in patients with intracranial involvement, primarily abscesses requiring craniotomy. These discrepancies suggest that the presence of intracranial extension is not necessarily an absolute predictor of mortality, but rather that the specific type of involvement -vascular thrombosis versus parenchymal abscess-largely dictates prognosis.

Age was identified as the sole independent prognostic factor for mortality (p=0.020). Conversely, traditional variables such as gender, uncontrolled DM, or haematological malignancy did not reach statistical significance in our multivariate analysis. However, other studies have reported higher mortality in males and patients with type 2 DM or orbital involvement (26). These variations highlight the multifactorial complexity of RCM prognosis, in which age, immunological recovery capacity, the timing of treatment and the anatomical extent interact. Furthermore, the association between RCM and solid tumours requires particular attention. Although less common than haematological malignancies, the increasing use of aggressive chemotherapy and immunotherapy in patients with solid tumours may create a new susceptibility niche that clinicians must recognise early.

With an overall mortality rate of 66.7%, our results are consistent with those reported in the literature, which reflect the high lethality of this infection (14 , 18 , 27 - 29). This rate is likely influenced by the advanced age and high prevalence of malignancies in our cohort. Based on these findings, we offer several recommendations. A high index of suspicion is mandatory in immunocompromised patients presenting with sinonasal or orbital symptoms. Empirical treatment should be initiated immediately upon clinical suspicion, without awaiting microbiological confirmation. Our results reinforce the importance of early diagnosis and timely surgical debridement, tailored sequentially to disease severity, as fundamental to improving prognosis. Equally vital is the aggressive management of the underlying immunosuppression. Finally, a coordinated, multidisciplinary approach integrating infectious diseases, oral and maxillofacial surgery, and otolaryngology is essential.

This study has limitations inherent to its retrospective design and single-centre nature. However, reports from Spain are scarce and limited to isolated cases (12 , 13 , 27). To the best of our knowledge, no recent, comprehensive series has specifically addressed RCM within the Spanish population. Adherence to a strict institutional protocol ensured consistency of therapeutic decisions over the 15-year period. Regarding surgical management, it should be noted that patients who were given antifungals alone may have been clinically unstable or have had a more advanced infection, which limits the validity of the survival comparison. Future improvements in outcomes will likely depend on earlier diagnosis, novel antifungal agents, and the broader implementation of standardised multidisciplinary protocols (30).

## Conclusions

RCM remains a rare but devastating opportunistic infection in our clinical setting, characterised by significant morbidity and high mortality, particularly among immunocompromised patients with haematological malignancies, solid tumours, or uncontrolled DM. Our findings underscore that early diagnosis and prompt treatment according to standardised multidisciplinary protocols, are pivotal for improving patient outcomes. This study provides critical data on the Spanish healthcare context, reinforcing the urgent need to disseminate and implement institutional guidelines that ensure comprehensive and timely management. The primary prognostic determinants identified -control of underlying immunosuppression, patient age, and early surgical intervention- highlight the necessity of an aggressive therapeutic strategy. Further multicentre studies are needed to validate these findings and optimise treatment algorithms.

## Figures and Tables

**Table 1:** Table Clinical manifestations and radiological findings (n=36).

		N (%)
Clinical manifestation*	Periorbital cellulitis/ophthalmoplegia	31 (86.1%)
	Paranasal sinusitis	17 (47.2%)
	Neurological symptoms	13 (36.1%)
	Palatal necrosis	9 (25%)
Radiological finding*	Paranasal sinusitis	35 (97.2%)
	Cellulitis (periorbital or nasomaxillary)	24 (66.7%)
	Intracranial extension	4 (11.1%)
	Bony erosion of facial skeleton	3 (8.3%)

*Some patients presented with two or more characteristics.

**Table 2:** Table Mortality according to type of treatment (n=36).

Treatment received	Deaths/n	Mortality (% of n)	pvalue
Antifungals alone	9/9	100%	<0.001*
Antifungals + surgical treatment	15/27	55.6%	

*Statistical significance p<0.05.

**Table 3:** Table Prognostic factors for mortality (n=36).

Variable		Deaths/n	Mortality (% of n)	pvalue
Gender	Female	7/12	58.3%	0.479
	Male	17/24	70.8%	
Age	≤55 years	4/11	36.4%	0.020*
	>55 years	20/25	80%	
Haematologic malignancy	Present	14/22	63.6%	1.000
	Absent	9/14	64.3%	
Solid tumour	Present	6/6	100%	0.079
	Absent	18/30	60%	
Uncontrolled DM	Present	4/8	50%	0.397
	Absent	20/28	71.4%	

*Statistical significance p<0.05.

**Table 4:** Table Mortality according to type of surgical treatment (n=27).

Type of surgery	Deaths/n	Mortality (% of n)	pvalue
ESS only	5/8	62.5%	0.858
Maxillectomy ± ESS	5/9	55.6%	
Orbital exenteration/nasal amputation ± ESS	1/3	33.3%	
Maxillectomy/exenteration/craniotomy ± ESS	4/7	57.1%	

4

## Data Availability

The data supporting the findings of this study are available from the corresponding author upon reasonable request.
